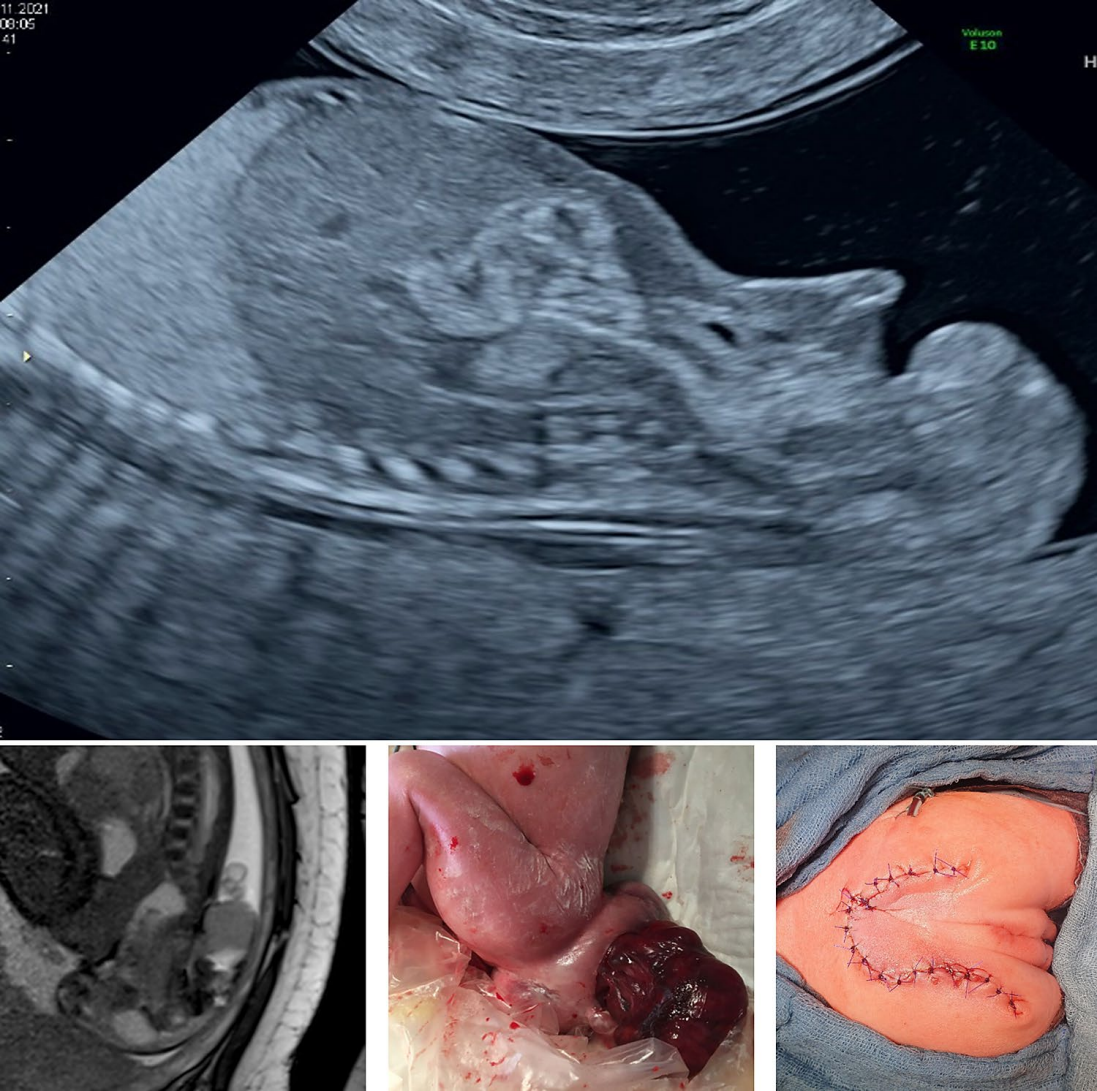# Exceptionally early diagnosis of fetal sacrococcygeal teratoma in first trimester ultrasound

**DOI:** 10.1007/s00404-022-06721-y

**Published:** 2022-08-03

**Authors:** Tobias Spingler, Cornelia Wiechers, Justus Lieber, Karl Oliver Kagan

**Affiliations:** 1 Department of Obstetrics and Gynaecology, University of Tuebingen, Calwerstrasse 7, 72076 Tübingen, Germany; 2 Department of Neonatology, University of Tuebingen, Calwerstrasse 7, 72076 Tübingen, Germany; 3 Department of Pediatric Surgery and Pediatric Urology, University of Tuebingen, Hoppe-Seyler-Str. 3, 72076 Tübingen, Germany

A 33-year-old primigravida was referred to us at 13 weeks’ gestation for a suspected fetal sacrococcygeal teratoma (SCT). Our ultrasound examination revealed a solid mass protruding from the external surface of the fetal coccyx, which was consistent with an exophytic SCT. The largest diameter was 1.5 cm with increased vascular perfusion. The crown rump length was 84 mm, and the nuchal translucency thickness was 2.2 mm. The fetus showed no signs of volume overload. Due to the unusually early diagnosis of this lesion, the prognosis was uncertain. The couple decided to continue the pregnancy. At 23 weeks, the diameter of the SCT was 5 cm and it increased to 7 cm at 31 weeks. Up to this point, there were no signs of fetal cardiac dysfunction on ultrasound. However, at 31 weeks, polyhydramnios developed. An MRI at 34 weeks’ gestation was consistent with an Altmann type II SCT with extra-fetal location composed of cystic and solid components. The size was 8 × 6 × 6 cm. At 34 weeks, the umbilical artery pulsatility index increased above the 95th centile, the other Doppler parameters were normal. Furthermore, the patient developed premature contractions with shortening of the cervix. After the administration of corticosteroids, a primary cesarean section was performed. A viable preterm female infant was delivered with an APGAR of 8/7/9, umbilical artery pH of 7.32, and a birth weight of 2200 g. The entire SCT was resected on the second day of life by pediatric surgery. Pathology revealed a 12 × 9x3 cm immature, type I (Gonzalez-Crussi) SCT. The neonate was discharged after 13 days.

SCTs are the most common congenital tumor in newborns with an approximately 4:1-female-to-male prevalence. Those with an external location, very little to no internal component, and no signs of cardiac stress have a favorable prognosis [3]. A diagnosis in the first trimester is exceptionally rare [1, 2].